# Genome Sequences of Three Novel Bunyaviruses, Two Novel Rhabdoviruses, and One Novel Nyamivirus from Washington State Moths

**DOI:** 10.1128/genomeA.01668-16

**Published:** 2017-02-16

**Authors:** Negar Makhsous, Ryan C. Shean, David Droppers, Jonathan Guan, Keith R. Jerome, Alexander L. Greninger

**Affiliations:** a Department of Laboratory Medicine, University of Washington, Seattle, Washington, USA; b Washington Butterfly Association, Seattle, Washington, USA; c Vaccine and Infectious Disease Division, Fred Hutchinson Cancer Research Center, Seattle, Washington, USA

## Abstract

We report draft genome sequences of three novel bunyaviruses, two novel rhabdoviruses, and one novel nyamivirus identified metagenomically from 10 moths in Washington state.

## GENOME ANNOUNCEMENT

Lepidoptera is a diverse order of insects with more than 180,000 species known throughout the world. In addition to being pollinators and food for other wildlife, some species act as serious agricultural pests (1). Many species can be found in suburban backyards, in close contact with people. In order to find new viruses in this undersampled order, we performed metagenomic next-generation sequencing (mNGS) on 10 moths across eight species captured in a suburban neighborhood north of Seattle, Washington. The eight species of moths sequenced included *Noctua pronuba*, *Tyria jacobaeaea*, the timber pest *Orgyia pseudotsugata* (two sampled), and the apple pests *Pasiphila rectangulata*, *Carcina quercana*, *Plutella xylostella*, *Choristoneura rosaceana*, and *Cydia pomonella* (two sampled) (1).

Total RNA was extracted from moths after bead-beating using the Zymo ZR viral RNA kit and 0.45 µM filtering followed by DNase treatment. Nextera XT libraries were constructed from double-stranded cDNA and amplified for 16 to 18 cycles of PCR (2–4). Sequencing reads were quality and adapter-trimmed, *de novo* assembled using SPAdes v3.8, and analyzed using HHPred (5, 6).

From one *Orgyia pseudotsugata* moth, an 11,308 nucleotide contig with five open reading frames (ORFs) was assembled corresponding to the novel rhabdovirus Orgi virus SP1. The L polymerase protein aligned 45% by amino acid to the Wuhan Fly Virus 2 (7). Sequencing of an additional *Orgyia pseudotsugata* moth yielded an 11,178 nucleotide contig (SP2) that aligned 99.6% by nucleotide to the original Orgi virus SP1. An additional 11,238 nucleotide contig with five ORFs was assembled from this sample corresponding to the novel rhabdovirus Gata virus M4. Its RdRp aligned closest to the Orgi virus at 47% by amino acid.

From the first *Cydia pomonella* sequenced, three segments corresponding to the RdRp, glycoprotein, and nucleocapsid/ORFIV segments of Mothra bunyavirus JG1 were assembled. Interestingly, sequencing of an additional *Cydia pomonella* revealed 170× coverage of the Mothra bunyavirus RdRp and nucleocapsid segments but no reads to the glycoprotein segment. The RdRp of the two Mothra bunyaviruses shared 97.3% amino acid identity and aligned 28% by amino acid to the RdRp Uukuniemi virus (AIU95041) isolated from *Ixodes ricinus* from the Czech Republic (8).

From the *Choristoneura rosaceana* library, 58,770 reads aligned to the three segments totaling 11,774 nucleotides of the Pidgey bunyavirus M6 genome. The RdRp of Pidgey bunyavirus aligned 29% by amino acid to the RdRp of the Rukutama virus isolated from *Ixodes uriae* in Russia (9).

From the *Pasiphila rectangulata* moth, three segments from a novel bunyavirus were assembled ranging from 6× to 14× coverage. The Seattle Prectang virus RdRp aligns 42% by amino acid to the Wuchang Cockroach Virus 1 from *Blatella germanica* (7). An additional 9,591 nucleotide contig with five ORFs corresponding to a novel mononegavirales member was assembled. The Orinoco virus UW1 RdRp aligned 33% by amino acid to the Midway nyavirus, while the closest glycoprotein HHPred matches were all herpesviral gB glycoproteins and the closest nucleoprotein HHPred match was Borna disease virus (10, 11). Based on phylogenetic comparisons, Orinoco virus would appear to constitute a likely novel genus within the *Nyamiviridae* family.

### Accession number(s).

These genomes have been deposited at DDBJ/ENA/GenBank under the accession numbers KX257488 (Orinoco virus UW1), KX852389 to KX852391 (Pidgey bunyavirus M6), KX272883 to KX272885 and KY293690 to KY293691 (Mothra bunyavirus JG1), KX272880 to KX272882 (Seattle Prectang UW1), KX852388 (Gata virus M4), and KX852386 to KX852387 (Orgi virus SP1, SP2).
